# A comprehensive overview of the cystic fibrosis on the island of São Miguel (Azores, Portugal)

**DOI:** 10.1186/s12887-019-1903-y

**Published:** 2020-01-03

**Authors:** Joana Rosa, Patrícia Gaspar-Silva, Paula Pacheco, Conceição Silva, Cláudia C. Branco, Barbara S. Vieira, Alexandra Carreiro, Juan Gonçalves, Luisa Mota-Vieira

**Affiliations:** 1 Pediatric Department, Hospital do Divino Espirito Santo de Ponta Delgada EPER, 9500-782 Ponta Delgada São Miguel Island, Azores, Portugal; 2 Centre for Human Genetics, Instituto Nacional de Saúde Dr. Ricardo Jorge, Lisbon, Portugal; 3 Molecular Genetics and Pathology Unit, Hospital do Divino Espirito Santo de Ponta Delgada EPER, São Miguel Island, Azores, Portugal; 4 Faculty of Sciences, BioISI - Biosystems & Integrative Sciences Institute, University of Lisboa, Lisbon, Portugal; 5 Azores Genetics Research Group, Instituto Gulbenkian de Ciência, Oeiras, Portugal; 6 Unidade de Saúde Pública da Unidade de Saúde da Ilha de São Miguel, São Miguel Island, Azores, Portugal; 7 Pneumology Department, Hospital do Divino Espirito Santo de Ponta Delgada EPER, São Miguel Island, Azores, Portugal

**Keywords:** Cystic fibrosis, Cystic fibrosis Transmembrane conductance regulator, Genotype-phenotype analysis

## Abstract

**Background:**

Early diagnosis and treatment are improving significantly the quality of life of patients with cystic fibrosis (CF). This recessive disease is caused by a great variability of mutations in the CF transmembrane conductance (*CFTR*) gene, whose spectrum and frequency can be different across populations.

**Methods:**

We performed a retrospective cross-sectional study of CF patients from the island of São Miguel (Azores, Portugal) through a clinical, genealogical, genetic and epidemiological investigation. The clinical course of patients was analyzed as a whole and according to their genotype.

**Results:**

We identified 14 CF patients within a 23-year period, corresponding to a cumulative incidence of 1:3012 births, being three of them born from consanguineous unions. Genetic analysis revealed three *CFTR* genotypes: p.[Ser4Ter];[Gln1100Pro] was present in one patient with a less severe phenotype (1/14); c.[120del23];p.[Phe508del], a very rare one (2/14); and p.[Phe508del];[Phe508del] in the remaining patients (11/14). Clinically, respiratory infections (8/14) and growth failure (6/14) were the most common initial manifestations. All patients presented pancreatic dysfunction, with 21.4 and 100% of them showing endocrine and exocrine insufficiency, respectively. As expected, patients with severe phenotype were homozygous for p.Phe508del and had the lowest value of body mass index.

**Conclusions:**

The present study demonstrated that São Miguel Island has an increased incidence of CF when compared to recent Portuguese data (1:7500 live births). It also allowed a comprehensive overview of CF in São Miguel, improving medical practice along with genetic counselling and creating opportunities for genotype-targeted therapies.

## Background

Cystic fibrosis (CF; OMIM #219700, ORPHA:586) is an autosomal recessive genetic disease, caused by loss-of-function mutations in the CF transmembrane conductance gene (*CFTR*; OMIM *602421) [1, 2]. Located on 7q31.2, *CFTR* codes for an epithelial chloride transporter, and has over 2075 mutations reported [3]. The most common mutation is the *CFTR*:c.1521_1523delCTT (p.Phe508del), present in 85.8% of CF patients worldwide [4], with a higher frequency reported in Northern Europeans [5]. The incidence of CF in Portugal is estimated at 1 in 6000 newborns [2] but, in a three-year (end of 2013 to 2016) CF pilot study conducted in the scope of the national newborn screening program, this value was 1 in 7500 newborns [6]. Both values are lower than the ones observed in European Union population: 1 in 2000–3000 newborns [7]. The pilot study, conducted in Portugal, allowed the implementation of a cystic fibrosis neonatal screening program since December 2018 at a national level. Recently, *CFTR* mutations are classified into seven classes (class IA, IB, II-VI), according to the mechanisms by which they produce quantitative or qualitative changes in CFTR function and to the corrective treatment predicted by new drugs available for precision medicine [8–11].

CF is a multisystemic disease affecting mainly lungs, digestive system and sweat glands. Although there is a variable clinical spectrum, progressive lung disease and pancreatic insufficiency are the main features [1]. Recurrent sino-pulmonary infections are one of the main challenges in CF, requiring a mounting use of antibiotics. This aspect, along with improved nutrition and new therapies, is responsible for the increase in survival rates in these patients and alters the antimicrobial susceptibility [4]. The most common pathogens of recurrent infections are *Pseudomonas aeruginosa, Staphylococcus aureus, Haemophilus influenza*, *Burkholderia spp.* and *Mycobacteria spp.* [12].

The emergence of therapies targeting specific mutations or mutation classes, a consequence of the clinical and genetic heterogeneity of CF, urges the study of this disease in different populations as a way to positively impact health care in CF. To that end we investigated an Atlantic insular CF population in terms of epidemiological, clinical, microbiological and genetic aspects, allowing the characterization of the mutational spectrum of *CFTR* on the Azorean island of São Miguel (Portugal) and its association with phenotypic manifestations.

## Methods

### Ethics statement and study design

The present investigation follows the international ethical guidelines and was approved by the board of the Hospital do Divino Espirito Santo de Ponta Delgada (HDES) after a favorable report by the Health Ethics Committee (Ref. HDES/CES/2016/382). This hospital is the only one serving the São Miguel Island, the most populated of the Azores, an archipelago composed by nine islands in the North Atlantic Ocean. São Miguel has 137,856 inhabitants, corresponding to 55.9% of Azores population (Portugal Census, 2011).

We conducted a retrospective cross-sectional study of patients with CF from the São Miguel Island, during the period of January 1st 2011 and December 31st 2016. A written informed consent was obtained from all patients if adults (≥18 years old), or their parents or legally authorized representatives (< 18 years old). If desired, they had the right to decline or withdraw at any stage of the study without loss of care. Confidentiality was assured by codification and anonymization of data. The study also used a DNA bank of unrelated healthy blood donors from the São Miguel Island. This bank was established after approval by the Health Ethics Committee of HDES and follows the international ethical guidelines for sample collection, processing, and storage [13].

### Patients selection, clinical parameters and genealogy construction

We included all patients enrolled in the CF follow up appointment, performed by Departments of Pediatrics and Pulmonology of HDES, which constitutes a satellite unit of the CF reference center located at Centro Hospitalar Universitário Lisboa Norte (Portugal). The diagnosis was based on clinical features suggestive of CF and sweat chloride value (≥60 mmol/L) and/or two CF-causing *CFTR* mutations, in accordance to the Consensus Guidelines from the Cystic Fibrosis Foundation [14]. We excluded three patients with CFTR dysfunction that did not fulfil diagnostic criteria for CF, commonly known as CFTR-related disease [15].

Clinical information was obtained by review of paper-based and electronic medical records. The variables collected were: demographic parameters (gender, municipality of birth, consanguinity and family history) and clinical data (age at diagnosis, initial and additional symptoms, sweat chloride and fecal elastase-1, determined by quantitative conductivity method and immunoenzymatic assay respectively, body mass index, and forced expiratory volume in the first second, FEV_1_). The assessment of pulmonary function followed reference values of the European Coal and Steel Community, and the spirometry classification was based on the Global Initiative for Chronic Obstructive Lung Disease (GOLD) guidelines [16].

We also carried out a genealogical reconstruction study. The mother and/or father of patients were interviewed using a structured questionnaire that comprised the assessment of family history tracing back to the third generation (names, date of birth and birthplaces of patients, their parents and grandparents). In cases of suspected family history in more distant generations, a local genealogist carried out a computer-based genealogy reconstruction study using name, date of birth, and dates and parishes of marriages of maternal and paternal ancestors. These data allowed the evaluation of consanguinity and endogamy (grandparents from the same locality).

### *CFTR* mutation analysis

Genomic DNA of CF patients was extracted from peripheral blood lymphocytes, according to the QIAamp Blood mini kit (Qiagen) protocol. *CFTR* mutation analysis, based on the GenBank cDNA reference sequence NM_000492.3, was performed by three steps. First, we screened the 36 most common CF-causing mutations in Caucasians and the *CFTR* polymorphism Tn in intron 8, using a reverse dot-blot approach: INNO-LiPA CFTR19 and INNO-LiPA CFTR17 + Tn Update (Innogenetics). Second, we used PCR-DGGE (denaturing gradient gel electrophoresis) method for exons 3, 12, 13 and 20. Finally, if one or both CF-causing mutations were not identified, we direct sequenced 9 exons (1, 4, 5, 6, 14, 17, 19, 21 and 22) of the *CFTR* gene and their associated splice site introns. Together, this strategy allowed us to identify approximately 98% of the CF-causing mutations in the Portuguese population. In the case of the first patient with the c.120del23 mutation, all 27 exons, respective splice site introns, 5′ and 3′ UTRs and [TG]_m_T_n_ were analyzed previously by direct sequencing [17]. The pathogenicity of *CFTR* variants was based on clinical and biological data available on public databases (e.g., ClinVar, CFTR2, PubMed).

For CF-causing mutations frequency estimation in the São Miguel’s general population, we used a representative group composed by 469 DNA samples of unrelated healthy blood donors from the anonymized Azorean DNA bank described by Branco C. and Mota-Vieira, 2011 [13]. The genotyping was performed using three PCR allelic discrimination techniques, namely: 1) conventional amplification-refractory mutation system (ARMS) for c.1521_1523delCTT (p.Phe508del) and c.3299A > C (p.Gln1100Pro); 2) conventional PCR for the c.120del23; and 3) TaqMan® pre-designed SNP genotyping assay (Life Technologies) for the c.11C > A (rs397508173; p.Ser4Ter). The primers and PCR conditions were summarized in Additional file 1: Table S1.

### Microbiology analysis

The bacterial and fungal isolates were obtained through culture of sputum or bronchoalveolar lavage. The susceptibility bacterial phenotypes were tested in Vitek 2 system (bioMérieux, Inc.), according to the European Committee on Antimicrobial Susceptibility Testing (EUCAST) recommendations [18] applicable during the time period of the study.

### Statistical analysis

We performed a descriptive analysis using IBM® SPSS® Statistics version 25.0 (released 2017). The cumulative incidence at birth of CF in São Miguel, from 1994 to 2016, was estimated based on the total number of patients diagnosed at the HDES and of live births registered in the Azores Regional Statistics Service. We also evaluated the parental consanguinity through the ascending genealogy until the third generation, followed by the calculation of inbreeding coefficient (F), to measure the degree of inbreeding among children from consanguineous unions.

## Results

### Demographic characteristics of CF patients and incidence at the birth of the disease

We ascertained 14 CF patients (10 unrelated, two brothers and 2 second cousins) born on the island of São Miguel, during a 23-year period (from January 1994 to December 2016). Their demographic characteristics are summarized in Table 1 and Additional file 2: Table S2. The group was composed by 10 children/adolescents and four adults, being the male:female ratio 1:1. The median age at diagnosis was 0.2 years (0–11 years), except for patient Pt2 who was diagnosed by prenatal testing due to familial history of CF in a sibling (Pt5). At the end of the study, the mean age was 15.1 years (range 6.0–22.2), a value that does not include the deceased patient (Pt11).

**Table 1:** Demographic, clinical and laboratory data of CF patients from São Miguel, distributed by *CFTR* genotypes

Patient features	*CFTR* genotypes			
	Homozygous	Compound heterozygous		Total
	p.[Phe508del/ Phe508del]	c.120del23/ p.Phe508del	p.[Ser4Ter; Gln1100Pro]	
Demographic and familiar data				
Patient ID (n)	Pt1-Pt8, Pt10, Pt13, Pt14 (11)	Pt11, Pt12 (2)	Pt9 (1)	14
Gender (female:male ratio)	5:6	1:1	1:0	1:1
Family history (nb of patients)	5/11	1/2	0/1	6/14
Consanguinity (nb of patients)	3/11	0/2	0/1	3/14
Clinical data				
Age at diagnosis (years; median ± IQR)	0.2 ± 0.3	5.9 ± 0.0	4.2	0.2 ± 1.3
Initial symptoms (nb of patients)				
Prenatal testing	1/11	0/2	0/1	1/14
Meconium ileus	4/11	1/2	0/1	5/14
Respiratory infections	6/11	1/2	1/1	8/14
Growth failure	5/11	0/2	1/1	6/14
Additional symptoms (nb of patients)				
Respiratory infections	11/11	2/2	1/1	14/14
Exocrine pancreatic insufficiency	11/11	2/2	1/1	14/14
CF-related diabetes	1/11	1/2	0/1	2/14
Sweat chloride concentration (mmol/L; mean ± SD)	101.3 ± 18.3	94.5 ± 34.6	113	101.1 ± 19.2
BMI (kg/m^2^; mean ± SD)	17.7 ± 3.4	17.9 ± 1.8	20.8	18.0 ± 3.1
FEV_1_ª (% of predicted values)				
First value (mean ± SD)	98.2 ± 16.5	60.1 ± 0.7	65.9	89.8 ± 21.4
Last value (mean ± SD)	85.9 ± 26.4	59.3 ± 0.4	42.5	78.4 ± 27.1
Bacterial flora characteristics				
First isolation (age in years mean ± SD)				
*Pseudomonas aeruginosa*	4.8 ± 2.7	7.5 ± 6.3	4.5	5.2 ± 3.1
*Burkholderia cepacia*	5.7 ± 0.2	NA	6.3	5.9 ± 0.4
*Staphylococcus aureus*	3.1 ± 1.3	7.5 ± 4.9	4.0	3.8 ± 2.4
Exacerbations *per* year *per* patient (mean ± SD)	1.7 ± 1.0	2.1 ± 1.8	1.8	1.7 ± 1.0
Admissions *per* year *per* patient (mean ± SD)	0.3 ± 0.3	1.6 ± 2.6	0.3	0.5 ± 0.9

*nb* Number, *IQR* Interquartile range, *SD* Standard deviation, *NA* Not applicable. ªFirst and last values of FEV1 measured during the time of the study

We calculated the incidence at birth of CF in São Miguel from 1994 to 2016. According to the Azores Regional Statistics Service, the total number of births was 42,162 (mean: 1833/year). Therefore, the cumulative incidence of the disease was 1:3012 births for São Miguel.

### *CFTR* mutation spectrum and familial analysis

The *CFTR* mutation analysis of the 14 CF patients (28 alleles) revealed four different mutations (Table 2), being p.Phe508del the most frequent (24/28, 85.7%). This deletion was found in homozygosity in 11 (78.6%) patients, and in compound heterozygosity with c.120del23 in two (14.3%) patients, previously described in detail [17]. The remaining patient (Pt9) was a compound heterozygote for two rare point mutations: one nonsense (c.11C > A, p.Ser4Ter) and one missense (c.3299A > C, p.Gln1100Pro). We also estimated the frequency of these four *CFTR* mutations in São Miguel population (Table 2). In a representative sample, composed by 469 unrelated healthy blood donors (938 alleles), we detected only two mutations, both in heterozygosity: the p.Phe508del in seven individuals and the p.Ser4Ter in one individual, corresponding to a variant allele frequency of 0.7 and 0.1%, respectively.

**Table 2:** Characteristics and frequency of *CFTR* mutations found in CF patients and general population of São Miguel. The table also shows the mutation frequency reported in public databases (CFTR2 and gnomAD)

*CFTR* mutation			Predicted functional class	Clinical significance (ClinVar interpretation)	Mutation frequency				
					Mutation allele	São Miguel Island			Public database
Legacy name	cDNA (exon; (NM_000492.3)	Protein (NP_000483.3)				CF patients (%)		General population (%)	
120del23^a^	c.-12_10del23 or c.120del23 (ex 1)	NA	VI	Pathogenic^b^	del23	2	(7.1)	0 (0.0)	ND
S4X	c.11C > A (ex 1)	p.Ser4Ter	IB	Pathogenic	A	1	(3.6)	1 (0.1)	0.00010^c^
deltaF508del	c.1521_1523delCTT (ex 11)	p.Phe508del	II	Pathogenic	delCTT	24	(85.7)	7 (0.7)	0.69744 ^c^
Q1100P	c.3299A > C (ex 20)	p.Gln1100Pro	Uncertain	Pathogenic/Likely pathogenic	C	1	(3.6)	0 (0.0)	0.000004^d^

^a^This deletion affects the 5′ untranslated region upstream of exon 1, including the translation initiation codon (ATG); ^b^This variant is not registered in ClinVar, but its pathogenicity was demonstrated by functional studies. *NA* Not applicable, *ND* Not described; ^c^CFTR2 database [25]; ^d^gnomAD database [26]

The Azores settlement began in 1439 mainly with Portuguese individuals, but had contributions, to a less extent, from people with different genetic backgrounds (Jews, Moorish prisoners, African slaves, Flemish, French and Spaniards) [19–21]. Although this genetic diversity, the autosomal recessive nature of CF and the relatively closed society of São Miguel islanders, mainly in the twentieth century (and previous), allowed us to investigate the family history of the disease, consanguinity and endogamy. We observed a positive family history in six (42.9%) patients and a parental consanguinity in three (21.4%) cases. The ascending genealogies showed that the inbreeding coefficient (F) was 0.0625 for patient Pt10 (born from first cousin parents) and 0.0039 for patients Pt1 and Pt4 (each one born from third cousin parents). Concerning the grandparental endogamy, 50% of patients (7/14; Pt3, Pt4, Pt6, Pt7, Pt9, Pt13, Pt14) belong to families with complete endogamy (the four grandparents are from the same locality). The remaining 42.9% (6/14) have partial endogamy: 4 patients (Pt5, Pt10, Pt11, Pt12) have three grandparents from the same locality, and 2 patients (Pt1, Pt2) have only two grandparents. Regarding geographical distribution in São Miguel, the majority of the patients (6/14; 42.9%) was originated from Ribeira Grande, a municipality with 23.3% of the island’s population.

### Clinical data and *CFTR* genotype of CF patients

We analyzed the clinical course of CF patients as a whole and according to their genotype (Table 1). The clinical manifestations leading to diagnosis were respiratory infections (8/14; 57.1%), growth failure (6/14; 42.9%) and meconium ileus (5/14; 35.7%), being the last parameter only observed in patients homozygous or compound heterozygous for p.Phe508del. At diagnosis, all patients had elevated sweat chloride levels (mean: 101.1 mmol/L; range: 70–122 mmol/L) and lower fecal elastase-1 value (mean: 21.2 μg/g of stool; range: 1–84 μg/g, for a normal reference value > 200 μg/g). The highest value of sweat chloride and the lowest result of fecal elastase-1 were identified in patients with homozygous genotype (Additional file 2: Table S2).

Clinically, the respiratory system is one of the most affected in CF. The assessment of pulmonary function was estimated using FEV_1_ predicted value. At baseline, 42.9% (6/14) of patients had moderate impairment (FEV_1_ ≥ 50 and < 80%, predicted); the remaining had mild dysfunction (FEV_1_ > 80%, predicted). In the last evaluation registered, the number of patients with moderate impairment increased to 50.0% (7/14); the patient Pt9, with p.[Ser4Ter];[Gln1100Pro] genotype, has the most pronounced decrease in FEV_1_ value (23.4%). The mean rate of pulmonary exacerbations was 1.7 *per* year *per* patient with 0.5 hospital admissions *per* year *per* patient. Patient Pt11, deceased (18.8 years) during the time of the study, had the highest value (5.0 pulmonary exacerbations and 4.8 admissions *per* year; Additional file 2: Table S2).

Pancreatic insufficiency is the most common gastrointestinal complication. Endocrine pancreatic dysfunction was present in 21.4% (3/14) of subjects, being two (Pt12, Pt13) with CF-related diabetes and one (Pt6) with hyperglycemia observed in the post-prandial and fasting periods (Table 1 and Additional file 2: Table S2). Exocrine pancreatic insufficiency was observed in all cases. We also evaluated nutritional status during the course of the disease. After a follow-up of 6 years, two patients (Pt5, Pt8; 14.3%), homozygous for p.Phe508del, had the lowest body mass index-for-age, with values below − 2 z-score, according to the standards of the World Health Organization.

### Bacterial flora characteristics

The most common microorganisms isolated in sputum samples were *Staphylococcus aureus*, *Pseudomonas aeruginosa* and *Haemophilus influenzae*. All patients had *Staphylococcus aureus*, being the first isolation at the age of 3.8 ± 2.4 years. *Pseudomonas aeruginosa* is the most common pathogen associated with decline of lung function. We detected this bacterium in 100% of subjects with an age at the first isolation of 5.2 ± 3.1 years. Despite the hospital’s best practices to control infection, *Burkholderia cepacia* was present in 3/14 patients (Pt2, Pt5, Pt9; 21.4%), two of them were siblings (Pt2, Pt5), being the first isolation at the age of 5.9 ± 0.4 years. We also identified other microorganisms in sputum samples, such as fungi, which were observed in 92.9% of patients. The most common isolate was *Aspergillus spp.* (28.6%), followed by *Candida spp*. (21.4%).

## Discussion

Cystic fibrosis occurs in all ethnicities (~ 70,000 patients worldwide), but the majority of patients are Caucasian of European descent [4]. In the present study, we observed an increased incidence of CF in São Miguel (1:3012 live births) when compared to recent Portuguese data (1:7500 live births) [6]. Although not corresponding to the same period of time analyzed and having been applied different methods for the estimation of incidence, we found that the incidence in São Miguel is approximately 2.5 folds the incidence estimated for Portugal. Several factors may have contributed, either individually or together, to this high incidence, namely: small and stable population size of the island of São Miguel (137,856 inhabitants; 55.9% of Azores); relatively closed society, at least, in past century and previous; unions between related individuals (21.4% of CF patients have consanguineous parents); and/or high endogamy (complete or partial).

Cystic fibrosis has lifelong implications and early diagnosis and treatment can significantly improve quality and expectancy of life. Recently, the knowledge of CF-causing mutations and their associated phenotypes has gained increased importance due to the blooming of targeted drugs for mutation specific treatment. However, the course from genotype to phenotype, and vice-versa, is not always easy to establish [22]. In São Miguel, we identified three *CFTR* mutated genotypes: p.[Ser4Ter];[Gln1100Pro], a new one; c.[120del23];p.[Phe508del], a very rare; and p.[Phe508del];[Phe508del], the most common genotype found in CF patients worldwide. To the best of our knowledge, the p.[Ser4Ter];[Gln1100Pro] genotype is neither described in literature nor registered in genetic and genomics databases. Indeed, both mutations are very rare and were described in only few CF patients having different genetic backgrounds, as summarized in the Additional file 3: Table S3. The p.Ser4Ter belongs to the functional CFTR class IB; however, the p.Gln1100Pro is only classified according to its pathogenicity as pathogenic / likely pathogenic (ClinVar). It will be interesting to investigate the cellular phenotype of the CFTR carrying the p.Gln1100Pro. Clinically, our patient (Pt9) had at initial presentation respiratory infections along with growth failure; subsequently, she presented exocrine pancreatic insufficiency, recurrent respiratory infections, and *Pseudomonas aeruginosa* chronic infection since 4 years-old. Together, the features observed in the course of the disease allowed us to suggest a less severe phenotype correlated to the p.[Ser4Ter];[Gln1100Pro] when compared to CF patients homozygous for the p.Phe508del mutation.

The very rare c.[120del23];p.[Phe508del] genotype, was identified in two patients (Pt11, Pt12), previously described [17]. As far as we know, the c.120del23 deletion was only reported in a Brazilian CF patient with African and Portuguese ancestry [23]. According to functional studies of *CFTR* gene containing this deletion (functional CFTR class VI), which involves the ATG of translation start codon, the N-truncated proteins produced can reach the cell membrane; however, they are unstable and have a reduced Cl- channel activity [17]. The two patients from the island of São Miguel have a clinical heterogeneity. The deceased female patient Pt11 (18.8 years), a gender associated with higher probability of death [24], had at initial presentation a suspected (but not confirmed) meconium ileus. Over the years, she presented a significant pulmonary impairment (bronchial obstruction and bronchiectasis) with a rapid decline of pulmonary function along with multiple exacerbations and admissions, mainly in the last 2 years of life. The other patient (Pt12) has a less severe phenotype, when compared with the patient Pt11. Indeed, he was only diagnosed at 11 years-old due to recurrent respiratory infections with bronchiectasis. He also has the most marked decline of FEV_1_ and CF related diabetes mellitus. The mentioned Brazilian CF patient with the c.[120del23];p.[Ser4Ter] genotype has a severe phenotype [23] and, interestingly, the two mutations were found separately in our CF group: c.[120del23] in patients Pt11 and Pt12, and p.[Ser4Ter] in the Pt19.

Finally, as expected, in CF patients from the island of São Miguel, we also found the most common *CFTR* genotype: p.[Phe508del];[Phe508del]. This mutation was present in 85.7% (24/28) of the alleles, a frequency higher than the one (79%) observed in the pilot study for CF newborn screening program conducted in Portugal. We hypothesize that consanguinity may contribute to this relatively high value, since 21.4% (3/14) patients are born from parents with a biological relationship. Moreover, the p.Phe508del frequency also corroborates with the higher CF incidence observed in this island.

The current work has some limitations. First, our study had a retrospective design and clinical information could have been missed. Second, due to the small number of CF patients, we were unable to perform a deeper genotype-phenotype analysis. Third, it should not be representative of the Azorean Archipelago, since the other eight small islands, which have 44.1% of the population, were not included. Moreover, although the Azorean population is, to a great extent, of Portuguese origin, the islands had a differential settlement with people from diverse genetic backgrounds. However, this study has at least three advantages. First, the island of São Miguel has only one hospital and all patients are followed in the same unit. Second, the study includes practically all of the island’s diagnosed cases that meet the inclusion criteria during a 23-year period (from 1994 to 2016). Finally, it is the first report on CF patients and *CFTR* mutations in the island of São Miguel.

## Conclusions

The present study demonstrated that São Miguel Island has an increased incidence of CF when compared to recent Portuguese data. It also allowed a better characterization of this recessive disease in an Atlantic island, improving medical practice along with genetic counselling and creating opportunities for mutation specific treatment.

## Supplementary information

**Additional file 1: Table S1.** Primers and PCR conditions for genotyping *CFTR* mutations in the general population of São Miguel.

**Additional file 2: Table S2.** Additional demographic, clinical characteristics and genetic data of each CF patient.

**Additional file 3: Table S3.** Genotypes and genetic backgrounds of CF patients with the p.Ser4Ter, p.Gln1100Pro or c.120del23, found in the literature and in the ClinVar database [27–35].

## Data Availability

The datasets used and/or analysed during the current study are available from the corresponding author on reasonable request.

## Abbreviations

ARMS: Conventional amplification-refractory mutation system
CF: Cystic Fibrosis
CFTR: CF transmembrane conductance
DGGE: Denaturing gradient gel electrophoresis
FEV_1_: Forced expiratory volume in the first second
HDES: Hospital do Divino Espirito Santo de Ponta Delgada
PCR: Polymerase chain reaction
Pt: Patient
UTR: Untranslated region
