# Supplementary material for: A comprehensive overview of the cystic fibrosis on the island of São Miguel (Azores, Portugal)
Source: BMC Pediatr. 2020 Jan 3;20:2. doi: 10.1186/s12887-019-1903-y (PMC6942372; doi:10.1186/s12887-019-1903-y)
Supplement: Supplementary file 1 — Additional file 1: Table S1. Primers and PCR conditions for genotyping CFTR mutations in the general population of São Miguel. [file 12887_2019_1903_MOESM1_ESM.docx]

| **Table S1** Primers and PCR conditions for genotyping *CFTR* mutations in the general population of São Miguel. | | | | | | | | |
| --- | --- | --- | --- | --- | --- | --- | --- | --- |
| **Genotyping assay** | ***CFTR* mutations** | | **Primers*** | | | **Annealing temperature (AT)** | **Number of cycles (NC)** | **PCR mix reactions** |
| Conventional ARMS PCR | c.1521_1523delCTT (p.Phe508del) | | WT – 5’GTATCTATATTCATCATAGGAAACACCACA3’ | | | 54ºC | 30 | A |
|  |  |  | VF – 5’GTATCTATATTCATCATAGGAAACACCATT3’ | | | 54ºC | 35 |  |
|  |  |  | CR – 5’GACTTCACTTCTAATGATGATTATGGGAGA3’ | | |  |  |  |
|  | c.3299A>C (p.Gln1100Pro) | | WT – 5’CATTTTGTGTTTATGTTATTTGCA3’ | | | 52ºC | 30 |  |
|  |  |  | VF – 5’GACAAAAATCATTTCTATTCTCATTG3’ | | | 54ºC | 30 |  |
|  |  |  | CR – 5’GACAAAAATCATTTCTATTCTCATTT3’ | | |  |  |  |
| Conventional PCR | c.120del23 | | F – 5’CGTAGTGGGTGGAGAAAGC3’ | | | 56ºC | 35 | B |
|  |  |  | R – 5’CCTTTACCCCAAACCCAACC3’ | | |  |  |  |
| TaqMan® pre‑designed SNP | c.11C>A (rs397508173; p.Ser4Ter) | | The reaction mix and PCR conditions were performed according to manufacturer’s (Life technologies) instructions, on a 7500 Fast Real Time PCR System. | | | | | |
| **PCR conditions** | | | | | | | | |
| **Steps** | **Temperature** | **Duration** | | **Number of cycles** | **PCR mix reactions** | | | |
| Enzyme activation | 95ºC | 15 minutes | | 1 | 1. 100 ng of genomic DNA, 10 µM primers, 100 nM dNTPs (Promega), 15 nM MgCl_2_ (Qiagen), 5x Q Solution (Qiagen), 10x buffer (Qiagen), 2 U of HotStart Taq (Qiagen) and sterile H_2_O to final reaction volume of 30 μl. 2. 50 ng of genomic DNA, 10 µM primers, 100 nM dNTPs (Promega), 5x Q Solution (Qiagen), 10x buffer (Qiagen), 5 U of HotStart Taq (Qiagen) and sterile H_2_O to final reaction volume of 25 μl. | | | |
| Denaturation | 94ºC | 1 minute | | NC |  |  |  |  |
| Annealing | AT | 1 minute | |  |  |  |  |  |
| Extension | 72ºC | 1 minute | |  |  |  |  |  |
| Final extension | 72ºC | 10 minutes | | 1 |  |  |  |  |
| *WT, wild-type forward primer; VF, variant forward primer; CR, common reverse primer; F, forward primer; R, reverse primer; AT, Annealing Temperature; NC, Number of cycles | | | | | | | | |
