# Supplementary material for: A comprehensive overview of the cystic fibrosis on the island of São Miguel (Azores, Portugal)
Source: BMC Pediatr. 2020 Jan 3;20:2. doi: 10.1186/s12887-019-1903-y (PMC6942372; doi:10.1186/s12887-019-1903-y)
Supplement: Supplementary file 2 — Additional file 2: Table S2. Additional demographic, clinical characteristics and genetic data of each CF patient. [file 12887_2019_1903_MOESM2_ESM.docx]

| **Table S2** Additional demographic, clinical characteristics and genetic data of each CF patient. | | | | | | | | | | | | | | | | | | | | | | | | | | |
| --- | --- | --- | --- | --- | --- | --- | --- | --- | --- | --- | --- | --- | --- | --- | --- | --- | --- | --- | --- | --- | --- | --- | --- | --- | --- | --- |
| **Demographic data** | | |  | **Clinical characteristics of the disease** | | | | | | | | | | | | | | | |  | **Consanguinity and genetic data** | | | | | |
|  |  |  |  | Initial presentation | | | | |  | Additional manifestations | | | | | | |  | Biochemical markers | |  | Consanguinity | |  | *CFTR* gene | | |
| Patient ID | Gender | Municipality |  | Age at diagnosis   (years) | Respiratory infections | Meconium ileus | Growth failure | Prenatal diagnosis |  | Respiratory infections | Exocrine pancreatic  insufficiency | Diabetes mellitus | FEV_1_ (% of predicted   value) | Body mass index (kg/m^2^) | Pulmonary   exacerbations/year | Admissions *per* year |  | Sweat chloride (mmol/L) | Fecal elastase-1 (µg/g) |  | Familiar history | Inbreeding coefficient of   child |  | Allele 1 | Allele 2 | |
| Pt1 | F | PDL |  | 0.3 | No | No | Yes | No |  | Yes | Yes | No | 95.0 | 15.1 | 0.17 | 0.17 |  | 109 | 15 |  | No | 0.008 |  | p.Phe508del | p.Phe508del | |
| Pt2 | M | RG |  | 0.0 | No | No | No | Yes |  | Yes | Yes | No | 96.9 | 13.4 | 1.83 | 0.17 |  | 118 | 5 |  | Yes | - |  | p.Phe508del | p.Phe508del | |
| Pt3 | M | VFC |  | 0.3 | Yes | No | Yes | No |  | Yes | Yes | No | 118.6 | 20 | 0.83 | 0.17 |  | 120 | 7 |  | Yes | - |  | p.Phe508del | p.Phe508del | |
| Pt4 | F | RG |  | 3.0 | Yes | No | Yes | No |  | Yes | Yes | No | NA | 14.9 | 2.67 | 0.83 |  | 122 | 1 |  | Yes | 0.004 |  | p.Phe508del | p.Phe508del | |
| Pt5 | M | RG |  | 0.2 | Yes | No | Yes | No |  | Yes | Yes | No | 59.0 | 15.1 | 3.50 | 1.00 |  | 80 | 1 |  | Yes | - |  | p.Phe508del | p.Phe508del | |
| Pt6 | M | POV |  | 0.7 | Yes | No | Yes | No |  | Yes | Yes | No^a^ | 43.6 | 16.6 | 2.00 | 0.17 |  | 80 | 1 |  | No | - |  | p.Phe508del | p.Phe508del | |
| Pt7 | M | RG |  | 0.2 | Yes | No | No | No |  | Yes | Yes | No | 71.9 | 18.7 | 1.67 | 0.33 |  | 92 | 2 |  | No | - |  | p.Phe508del | p.Phe508del | |
| Pt8 | M | RG |  | 0.0 | No | Yes | No | No |  | Yes | Yes | No | 104.8 | 15.6 | 0.67 | 0.00 |  | 107 | 60 |  | No | - |  | p.Phe508del | p.Phe508del | |
| Pt9 | F | NOR |  | 4.2 | Yes | No | Yes | No |  | Yes | Yes | No | 42.5 | 20.8 | 1.83 | 0.33 |  | 113 | 12 |  | No | - |  | p.Ser4Ter | p.Gln1100Pro | |
| Pt10 | F | PDL |  | 0.0 | No | Yes | No | No |  | Yes | Yes | No | 87.6 | 22.8 | 0.33 | 0.33 |  | 70 | 5 |  | Yes | 0.063 |  | p.Phe508del | p.Phe508del | |
| Pt11^b^ | F | VFC |  | 0.1 | No | Yes | No | No |  | Yes | Yes | No | 59.6 | 16.6 | 5.00 | 4.75 |  | 70 | 3 |  | Yes | - |  | p.Phe508del | c.120del23 | |
| Pt12 | M | PDL |  | 11.6 | Yes | No | No | No |  | Yes | Yes | Yes | 59.0 | 19.2 | MD^c^ | 0.00 |  | 119 | 84 |  | No | - |  | p.Phe508del | c.120del23 | |
| Pt13 | F | RG |  | 0.1 | No | No | Yes | No |  | Yes | Yes | Yes | 60.0 | 19.1 | 1.00 | 0.00 |  | 100 | 19 |  | No | - |  | p.Phe508del | p.Phe508del | |
| Pt14 | F | LAG |  | 0.0 | No | No | Yes | No |  | Yes | Yes | No | 121.1 | 23.8 | MD^c^ | MD |  | 116 | 82 |  | No | - |  | p.Phe508del | p.Phe508del | |
| F, female; M, male; LAG, Lagoa; NOR, Nordeste; PDL, Ponta Delgada; POV, Povoação; RG, Ribeira Grande; VFC, Vila Franca do Campo; NA, not applicable; ^a^Hyperglycemias observed in the post-prandial and fasting periods; ^b^Patient deceased at 18 years old; ^c^MD, Missing Data | | | | | | | | | | | | | | | | | | | | | | | | | |  |
