# Supplementary material for: A comprehensive overview of the cystic fibrosis on the island of São Miguel (Azores, Portugal)
Source: BMC Pediatr. 2020 Jan 3;20:2. doi: 10.1186/s12887-019-1903-y (PMC6942372; doi:10.1186/s12887-019-1903-y)
Supplement: Supplementary file 3 — Additional file 3: Table S3. Genotypes and genetic backgrounds of CF patients with the p.Ser4Ter, p.Gln1100Pro or c.120del23, found in the literature and in the ClinVar database [27–35]. [file 12887_2019_1903_MOESM3_ESM.docx]

| **Table S3** Genotypes and genetic backgrounds of CF patients with the p.Ser4Ter, p.Gln1100Pro or c.120del23, found in the literature and in the ClinVar database. | | | | |
| --- | --- | --- | --- | --- |
| **CF patients** | | | | **References** |
| *CFTR* genotype | | Genetic background/ Ethnicity | Number of patients |  |
| Allele 1 | Allele2 |  |  |  |
| p.Ser4Ter | p.Gln1100Pro | Portugal | 1 | Present study |
|  |  |  |  |  |
|  | p.Ser4Ter | MD | 3 | [28] |
|  |  |  |  |  |
|  | c.120del23 | Brazil | 1 | [23] |
|  |  |  | |  |
|  | p.Phe508del | Slovenia | 1 | [29] |
|  | p.Phe508del | Lebanon | 2 | [30] |
|  | p.Phe508del | MD | 4 | [28] |
|  |  |  |  |  |
|  | 2789+5G→A | Lebanon | 1 | [30] |
|  |  |  |  |  |
| p.Gln1100Pro | p.Gln1100Pro | Spain | 1 | [31] |
|  | p.Gln1100Pro | Brazil | 1 | [25] |
|  |  |  |  |  |
|  | p. Gly85Glu | Palestine | 1 | [32] |
|  |  |  |  |  |
|  | p.Phe508del | Portugal | 2 | [33] |
|  | p.Phe508del | Hispanic | 1 | [34] |
|  |  |  |  |  |
|  | p.Leu1077Pro | Australia | 1 | [35] |
|  |  |  |  |  |
|  | p.Asn1303Lys | Brazil | 1 | [25] |
|  |  |  |  |  |
| c.120del23 | p.Phe508del | Portugal | 2 | Present study, [17] |
|  | p.Ser4Ter | Brazil | 1 | [23] |
| MD, missing data. | | | | |
